# Predictive factors for the development of leukemia in patients with transient abnormal myelopoiesis and Down syndrome

**DOI:** 10.1038/s41375-021-01171-y

**Published:** 2021-03-03

**Authors:** Genki Yamato, Takao Deguchi, Kiminori Terui, Tsutomu Toki, Tomoyuki Watanabe, Takahiro Imaizumi, Asahito Hama, Shotaro Iwamoto, Daisuke Hasegawa, Takahiro Ueda, Tomoko Yokosuka, Shiro Tanaka, Ryu Yanagisawa, Katsuyoshi Koh, Akiko M. Saito, Keizo Horibe, Yasuhide Hayashi, Souichi Adachi, Shuki Mizutani, Takashi Taga, Etsuro Ito, Kenichiro Watanabe, Hideki Muramatsu

**Affiliations:** 1 Department of Hematology/Oncology, Gunma Children’s Medical Center, Gunma, Japan; 2 Department of Pediatrics, Gunma University Graduate School of Medicine, Gunma, Japan; 3 Children’s Cancer Center, National Center for Child Health and Development, Tokyo, Japan; 4 Department of Pediatrics, Hirosaki University Graduate School of Medicine, Aomori, Japan; 5 Department of Nutritional Science, Faculty of Psychological and Physical Science, Aichi Gakuin University, Aichi, Japan; 6 Department of Advanced Medicine, Nagoya University Hospital, Nagoya, Japan; 7 Department of Hematology and Oncology, Children’s Medical Center, Japanese Red Cross Nagoya First Hospital, Nagoya, Japan; 8 Department of Pediatrics, Mie University Graduate School of Medicine, Mie, Japan; 9 Department of Pediatrics, St Luke’s International Hospital, Tokyo, Japan; 10 Department of Pediatrics, Nippon Medical School, Tokyo, Japan; 11 Department of Hematology/Oncology, Kanagawa Children’s Medical Center, Kanagawa, Japan; 12 Clinical Biostatistics, Kyoto University Graduate School of Medicine, Kyoto, Japan; 13 Department of Hematology and Oncology, Nagano Children’s Hospital, Nagano, Japan; 14 Department of Hematology/Oncology, Saitama Children’s Medical Center, Saitama, Japan; 15 Clinical Research Center, National Hospital Organization Nagoya Medical Center, Nagoya, Japan; 16 Institute of Physiology and Medicine, Jobu University, Gunma, Japan; 17 Department of Human Health Sciences, Kyoto University, Kyoto, Japan; 18 Department of Pediatrics and Developmental Biology, Tokyo Medical and Dental University, Tokyo, Japan; 19 Department of Pediatrics, Shiga University of Medical Science, Otsu, Japan; 20 Department of Hematology and Oncology, Shizuoka Children’s Hospital, Shizuoka, Japan; 21 Department of Pediatrics, Nagoya University Graduate School of Medicine, Nagoya, Japan

**Keywords:** Acute myeloid leukaemia, Clinical trials

Transient abnormal myelopoiesis (TAM), also known as transient leukemia or transient myeloproliferative disorder, occurs in approximately 5–10% of neonates with Down syndrome (DS) and is characterized by the transient appearance of blast cells with megakaryoblastic and/or erythroblastic characteristics in the peripheral blood [1, 2]. Approximately 20% of TAM cases result in early death and 16–23% of survivors develop acute megakaryoblastic leukemia (AMKL) within 4 years [3–6]. A somatic *GATA1* gene mutation is shared by both TAM [7] and AMKL cells [8, 9].

Preceding studies have identified several risk factors associated with early death, including high white blood cell (WBC) count (≥100 × 10^9^/L), preterm delivery (<37 weeks), elevated direct bilirubin (≥5 mg/dL), hepatomegaly, ascites, and bleeding diatheses [3–6]. However, the definite clinical predictive indicators of AMKL onset in patients with TAM remain unclear. Here, we analyzed 167 TAM patients with DS who were enrolled in the TAM-10 prospective observational study conducted by the Japan Pediatric Leukemia/Lymphoma Study Group to determine the clinical characteristics of TAM and the predictive factors of leukemia development.

During 2011–2014, 167 neonates diagnosed with TAM were prospectively registered in the TAM-10 study (UMIN000005418, http://www.umin.ac.jp/ctr/index.htm). The details of the eligibility criteria, methods of flow cytometric minimal residual disease (FCM-MRD), *GATA1* mutations analysis, meta-analysis of DS-TAM clinical studies, and statistical analysis were described in Supplementary methods (Supplementary Tables 1–3).

The clinical characteristics and laboratory findings of 167 patients with TAM are summarized in Supplementary Table 4. The median (range) value of WBC was 38.3 (2.4–478.7) × 10^9^/L. Of the 167 patients, somatic *GATA1* gene mutations were confirmed in 163 (98%) patients (10 identified by next-generation sequencing [NGS] only). Patients with *GATA1* mutations identified only using NGS had significantly lower WBC counts (*P* < 0.001) and blast percentage (*P* < 0.001) than those with *GATA1* mutations identified by Sanger sequencing at diagnosis (data not shown). *GATA1* mutations were divided into high or low expression types according to the definition of a previous report (Supplementary Fig. 1) [10]. Details and expression type of *GATA1* mutations are described in Supplementary Table 5. FCM-MRD positivity (cutoff level, ≥0.1%) was detected at 1 and 3 months in 107 of 133 (80%) and 20 of 104 (21%) patients, respectively.

Sixty-eight of the 167 (41%) patients received some therapeutic interventions, including low dose cytarabine (LDAC) (*n* = 52), exchange blood transfusion (ET) (*n* = 20), and systemic steroid therapy (*n* = 30). The LDAC dose ranged from 1.0 to 1.5 mg/kg/day, and the median duration of treatment was 6 days (Supplementary Table 6). Details of systemic steroid therapy in 30 patients with TAM are summarized in Supplementary Tables 7 and 8. High-grade adverse events (grades 3 and 4) according to the CTCAE version 3.0 occurred in 52 patients who received LDAC and are described in Supplementary Table 9. High-frequency adverse events from LDAC therapy included neutropenia (65%), anemia (56%), thrombocytopenia (65%), and elevated blood bilirubin (56%). However, no patients died from adverse events with LDAC therapy.

The 4-year OS and EFS were 81% (95% confidence interval [CI]; 74.2–86.3%) and 65% (95% CI; 57.3–71.9%), respectively (Fig. 1A, B). Thirty-one of the 167 patients (19%) died, and their causes of death were described in Supplementary Table 10. Of these, early death (<9 months of age) occurred in 22 of 167 (13%) patients. Among the 138 patients without death events, 28 (20%) developed AMKL. The CI rate (CIR) of early death at 9 months was 17.6% (95% CI; 12.2–23.8%) and the rate of leukemia development at 4 years was 17.3% (95% CI; 11.9–23.5%) (Fig. 1C, D).

**Fig. 1:**
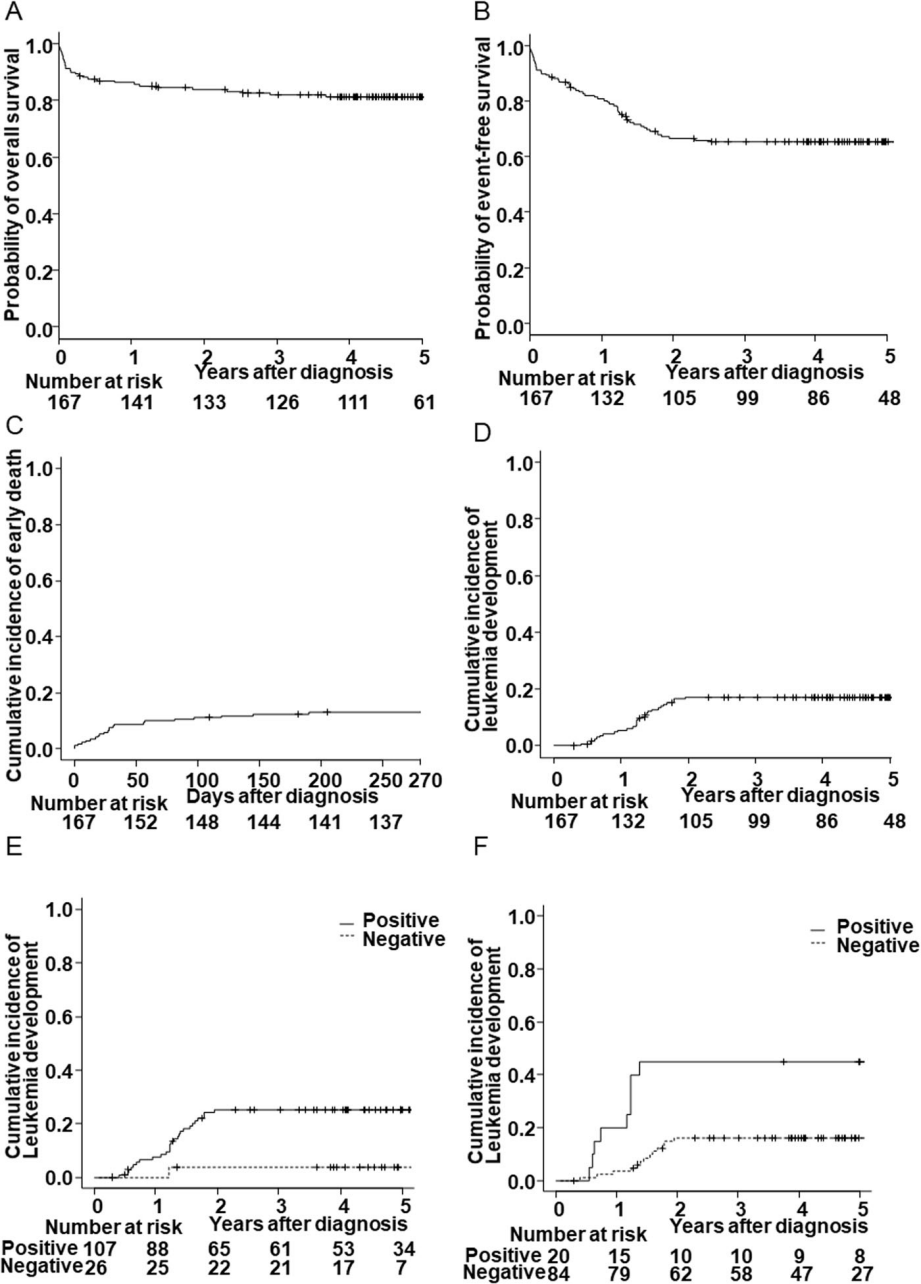
Outcomes of 167 patients with TAM and FCM-MRD analysis to predict leukemia development. Kaplan–Meier curves of overall survival (**A**) and event-free survival (**B**). Cumulative incidence rates of early death (**C**) and leukemia development (**D**). Cumulative incidence rates of leukemia development between TAM patients with and without FCM-MRD positivity at 1 month (positive, *n* = 107; negative, *n* = 26; CIR [95% CI] = 25.2% [17.3–33.9%] vs 3.8% [0.3–16.8%], *P* = 0.022) (**E**) and 3 months (positive, *n* = 20; negative, *n* = 84; CIR [95% CI], 45.0% [22.3–65.4%] vs. 16.0% [9.0–24.8%], *P* = 0.002) (**F**).

Univariable and multivariable analysis of the risk factors for early death are shown in Supplementary Table 11. The multivariable analysis identified the following independent risk factors for early death: a high WBC count (*P* = 0.002), systemic edema (*P* < 0.001), low birth weight (*P* = 0.037), systemic steroid therapy (*P* = 0.007), and elevated direct bilirubin (*P* = 0.007).

Leukemia development was significantly associated with FCM-MRD positivity at 1 month (*P* = 0.022) and 3 months (*P* = 0.002) (Fig. 1E, F). Furthermore, systemic steroid therapy was inversely associated with leukemia development (*P* = 0.008) (Supplementary Fig. 2), although 50% (15 of 30) of patients who received systemic steroid therapy died before leukemia development, reflecting their severe clinical conditions.

Univariable and multivariable analyses of the risk factors for leukemia development are shown in Supplementary Table 12. The multivariable analysis demonstrated that the positivity of FCM-MRD at 3 months and systemic steroid therapy were independent predictive factors for leukemia development.

In the subgroup of 36 patients with a high WBC count (≥100 × 10^9^/L), LDAC therapy significantly improved survival (*P* = 0.017) (Fig. 2A). However, there was no significant difference in the CIR of early death between patients with or without other therapeutic interventions, including ET or systemic steroid therapy (Supplementary Fig. 3).

**Fig. 2:**
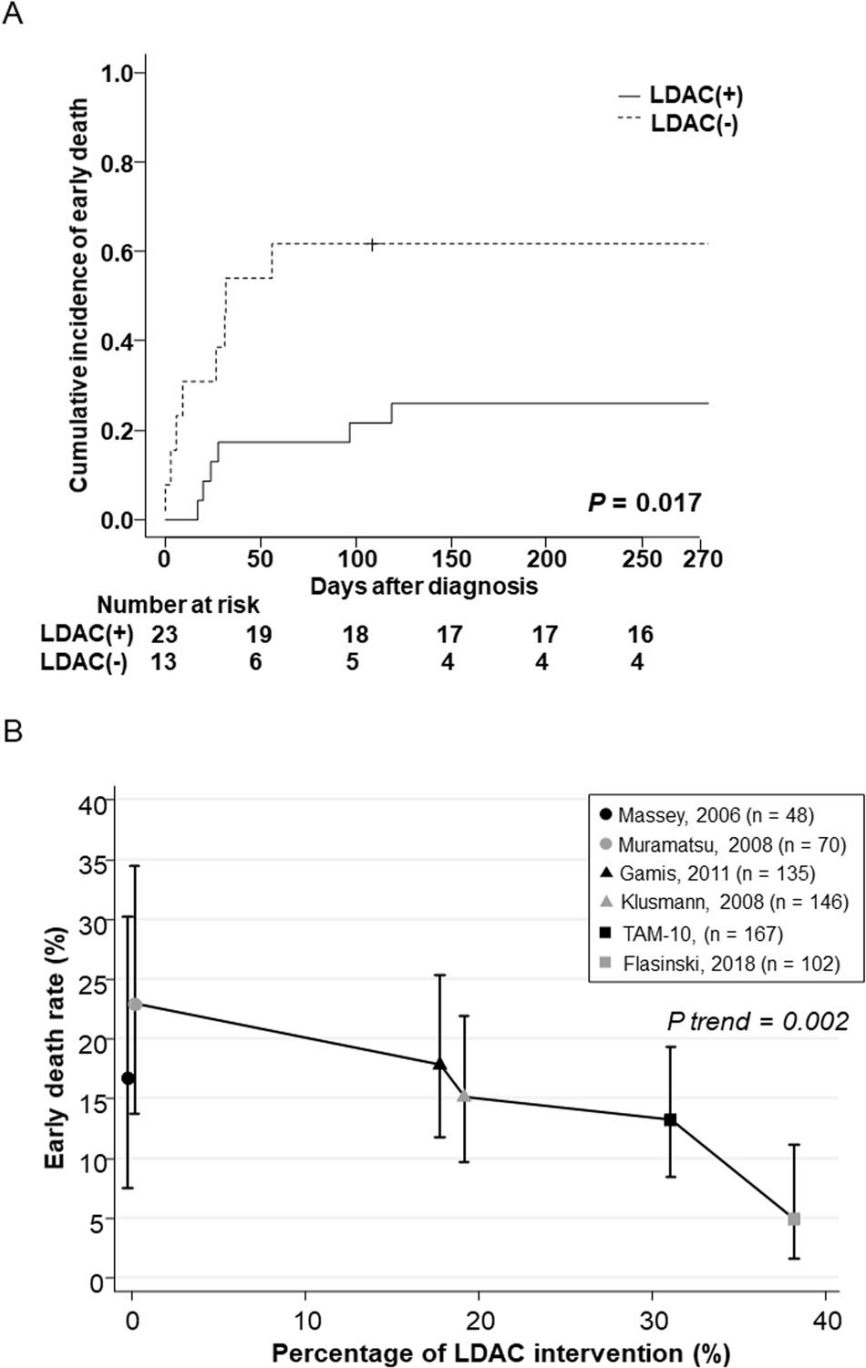
LDAC intervention is an indispensable therapy aimed at reducing the early death rate. **A** Subgroup analysis in patients with a high WBC count (≥100 × 10^9^ cells/L). The cumulative incidence rate of early death in 36 patients with a high WBC count indicated that LDAC therapy significantly improved survival (CIR [95% CI], 31% [13–50%] vs. 62% [29–83%], *P* = 0.017). **B** LDAC intervention was adversely associated with the early death rate. In the meta-analysis of large cohorts including this study and five other published studies, a higher proportion of patients receiving the LDAC intervention was significantly associated with a lower early death rate using the Cochran-Armitage test for trend.

We performed a meta-analysis of the current study and five previously reported cohorts of TAMs (Supplementary Table 13) [3–6, 11]. We compared WBC counts, the rate of early death, the CI of leukemia development, and the rate of interventions, including LDAC, ET, and systemic steroid therapy. Remarkably, the rate of LDAC intervention was adversely associated with the early death rate according to the Cochran-Armitage test for trend (*P* = 0.002, Fig. 2B). The cohorts without LDAC intervention [5, 6] reported higher early death rates; conversely, the cohorts with a higher percentage of LDAC intervention rates (31–38%) [11] (this study) reported lower early death rates. Meanwhile, there was no significant difference or trend in leukemia development among these cohorts.

TAM patients with life-threatening symptoms should be treated with LDAC [3, 4, 11, 12]. In this TAM-10 study, LDAC therapy significantly reduced the early death rates for patients with a high WBC count (≥100 × 10^9^/L) who are considered to be severe TAM patients (Fig. 2A). Furthermore, we confirmed the high safety profile of LDAC treatment and noted that no patients died of adverse events from LDAC therapy (Supplementary Table 9). These safety assessment results will allow us to safely broaden the threshold for LDAC intervention.

Meanwhile, the meta-analysis of this TAM-10 study and the other five reported large TAM cohorts (>40 cases) [3–6, 11] identified a significant trend between a higher rate of LDAC intervention and the reduction of early death rates (Fig. 2B). In particular, Flasinski et al. reported the highest rate of patients receiving therapeutic LDAC interventions (38%) and achieved a very low early death rate of 5%. In the TAM-10 study, we reported the second-highest rate of patients receiving therapeutic LDAC interventions (31%) and achieved the second-lowest early death rate (13%). These results suggest that LDAC therapy is an indispensable therapy for at least severe TAM patients, and more patients might be eligible for this treatment than previously considered.

Consistent with previous studies [3, 11], no clinical parameters predicted leukemia development in this study. However, we determined that FCM-MRD positivity at 3 months was a significant predictive indicator for leukemia development in the TAM-10 study cohort. Flasinski et al. also reported that being MRD-positive at week 12 was significantly associated with AMKL development [11]. These observations demonstrate the importance of FCM-MRD positivity for the prediction of leukemia development in patients with TAM. In this TAM-10 study, the FCM-MRD positivity cutoff value was set at 0.1%, but more sensitive MRD analyses can be achieved by using error-corrected NGS [13]. The clinical significance of NGS-MRD for TAM patients should be clarified in future studies.

Flasinski et al. reported that MRD-guided LDAC therapy for patients who were MRD-positive at eight and ten weeks failed to prevent AMKL development [11]. In this TAM-10 study, there was no significant difference in the incidence of AMKL between patients who had or had not been treated with LDAC; conversely, systemic steroid therapy showed a significant protective effect for leukemia development in the multivariable analysis (*P* < 0.001). An in vitro drug sensitivity test of 41 drug panels showed that the peripheral blood mononuclear cells (PBMCs) of five patients with TAM showed a higher sensitivity to cytarabine, trametinib, and dexamethasone than the PBMCs of healthy volunteers [14]. These results suggest that steroids may be a candidate for AMKL prophylactic intervention in patients with TAM. However, only 15 patients who were administered steroid therapy and survived were analyzed; therefore, well-designed clinical research studies need to be conducted on this subject in future.

Limitations of this study include the fact that the TAM-10 study was a prospective observational study, and the dose and duration of LDAC therapy were not uniform. Furthermore, the patients enrolled in the TAM-10 study had a higher frequency of congenital heart malformations (67%) and higher WBC counts than in a previous population-based DS registry study [15]. This study may have a cohort bias, owing to the inclusion of more symptomatic cases.

In summary, this TAM-10 study revealed that LDAC intervention is an indispensable therapy for reducing the early death rate and FCM-MRD is a useful marker for leukemia development in patients with TAM. Furthermore, systemic steroid therapy might suppress leukemia development. These results provide useful information for the design of future clinical studies to improve early mortality and AMKL incidence in patients with TAM.

## Supplementary information

Supplementary Information
