# Supplementary material for: Predictive factors for the development of leukemia in patients with transient abnormal myelopoiesis and Down syndrome
Source: Leukemia. 2021 Mar 3;35(5):1480–4. doi: 10.1038/s41375-021-01171-y (PMC8102190; doi:10.1038/s41375-021-01171-y)
Supplement: Supplementary file 1 — Supplementary Information [file 41375_2021_1171_MOESM1_ESM.docx]

## **Supplementary information**

**Supplementary Methods**

*Patients*

Between May 2011 and February 2014, 167 neonates (89 boys and 78 girls) diagnosed with TAM were prospectively registered in the TAM-10 study conducted by the JPLSG of the Japan Children’s Cancer Group. The TAM-10 study is registered with the UMIN Clinical Trials Registry (UMIN-CTR, URL: http://www.umin.ac.jp/ctr/index.htm), number UMIN000005418.

The eligibility criteria for the analysis were DS infants who were younger than three months and had circulating blast cells in the peripheral blood and non-DS infants who were younger than three months and had circulating blast cells in the peripheral blood that harbored somatic trisomy 21 and *GATA1* mutation. Patients with leukocytosis and peripheral blast cells as a result of infections or blood group incompatibility were excluded. Morphological diagnosis, flow cytometric analysis, and screening for mutations in *GATA1* as per the Central Review System of the TAM-10 study were recommended. Constitutional trisomy 21 was confirmed by the participating hospitals. The TAM-10 trial was a prospective observational study that did not require a protocolized therapeutic intervention. However, the TAM committee recommended low-dose cytarabine (LDAC) at 1.0 mg/kg/day × 7 days for patients with high WBC counts (≥100 × 10^9^/L) during the case enrollment period. Clinical data and sample collections in the clinical trials were approved by the institutional review boards of each participating institution, and written informed consent was obtained from the parents/guardians of all the study subjects. The present study was conducted as per the principles in the Helsinki Declaration and was approved by the ethical review board of the JPLSG. Moreover, the definition of the development of AMKL after TAM in this TAM-10 study is “once disappeared megakaryoblast is found after 90 days of age”.

*Flow cytometric analysis and minimal residual disease measurement*

Antibodies used for the diagnosis of TAM using flow cytometry are listed in **Supplementary Table 1,** and antibodies for the flow cytometric minimal residual disease (FCM-MRD) measurements are listed in **Supplementary Table 2**. The analysis was performed in eight colors, and at least 100,000 events were captured. The detection sensitivity was set at 0.1%. We analyzed FCM-MRD in 133 patients at 1 month and in 104 patients at 3 months for which samples were available. We compared 150 patients with (n = 104) and without FCM-MRD data at 3 months (n = 46), excluding 17 patients who died before 3 months of age, and found no significant difference between two groups in prognostic factors with early death, such as WBC counts, direct bilirubin level, gestational age, birth weight, and the presence of systemic edema **(Supplementary Table 3)**. Peripheral blood was incubated with the antibodies for 15 minutes at room temperature in the dark, and then red blood cells were lysed with FACS Lysing Solution (BD Bioscience, Franklin Lakes, USA) and washed once with Dulbecco’s phosphate buffered saline (Sigma-Aldrich, St Louis, USA). Antibody labeling measurements were performed using a FACS Canto II flow cytometer (BD Biosciences). FCM-MRD detection was performed with aberrant immunophenotypes determined during the diagnosis of each patient. The data were analyzed using the FACS DIVA software (BD Biosciences). At least 100,000 viable nucleated cells (up to 1,000,000) were analyzed in each sample, and MRD ≥ 0.1% was regarded as positive.

*GATA1 mutation analysis*

The *GATA1* mutations were analyzed by Sanger sequencing using genomic DNA and complementary DNA prepared from peripheral blood as described previously.^1^ Targeted next-generation sequencing was performed for patients in whom *GATA1* mutations were not detected by Sanger sequencing.^1^

*Meta-analysis of DS-TAM clinical studies*

We searched for cohorts with >40 TAM patients for the meta-analysis as follows. A systematic literature search was conducted by a research librarian using a combination of controlled vocabulary, when possible, and keywords as per the guidelines of Preferred Reporting Items for Systematic Reviews and Meta-Analyses (PRISMA). The PubMed database (<https://pubmed.ncbi.nlm.nih.gov/advanced/>) was searched for articles published from the time of its inception through July 2020. Terminologies that were searched included Down syndrome and representatives of different descriptions of TAM (e.g., “transient leukemia” OR “transient abnormal myelopoiesis” OR “transient myeloproliferative disorder”). We identified 499 articles written in English with text identified via an electronic database. Of these, 73 articles with only abstract, 166 case reports, and 84 review articles were excluded. Furthermore, 153 articles were excluded after evaluating the title. We carefully evaluated the abstracts and texts of the remaining 23 articles and selected five articles that reported cohorts with >40 TAM patients each.^2-6^

*Statistical analyses*

Overall survival (OS) and event-free survival (EFS) were estimated using the Kaplan–Meier method. Differences in survival were assessed using the log-rank test. The cumulative incidence (CI) for competing events was compared using the Gray test. The correlations between patients’ covariates and early death (<9 months of age) or leukemia development were evaluated using univariable and multivariable Fine-Gray proportional subhazards models. The following covariates were evaluated: sex, gestational age, birth weight, congenital heart disease, other congenital abnormalities, organ hemorrhage, systemic edema, WBC counts, blast percentage in peripheral blood, type of *GATA1* mutation, therapeutic interventions (LDAC, ET, and systemic steroid therapy), hepatomegaly, direct bilirubin, and the FCM-MRD at 1 and 3 months. The Cochran-Armitage test was used to examine trends in the relationship between the proportion of LDAC intervention and the early death rate in the meta-analysis. All the statistical analyses were performed using EZR software (version 1.36; Saitama Medical Center, Jichi Medical University, Saitama, Japan).^7^ In all analyses, *P* values were two-tailed, and *P* values < 0.05 were considered statistically significant.

References

1. Terui K, Toki T, Taga T, Iwamoto S, Miyamura T, Hasegawa D, et al. Highly sensitive detection of GATA1 mutations in patients with myeloid leukemia associated with Down syndrome by combining Sanger and targeted next generation sequencing. Genes Chromosomes Cancer. 2020;59:160-167.

2. Gamis AS, Alonzo TA, Gerbing RB, Hilden JM, Sorrell AD, Sharma M, et al. Natural history of transient myeloproliferative disorder clinically diagnosed in Down syndrome neonates: a report from the Children’s Oncology Group Study A2971. Blood. 2011;118:6752-6759.

3. Klusmann JH, Creutzig U, Zimmermann M, Dworzak M, Jorch N, Langebrake C, et al. Treatment and prognostic impact of transient leukemia in neonates with Down syndrome. Blood. 2008;111:2991-2998.

4. Massey GV, Zipursky A, Chang MN, Doyle JJ, Nasim S, Taub JW, et al; Children’s Oncology Group (COG). A prospective study of the natural history of transient leukemia (TL) in neonates with Down syndrome (DS): Children’s Oncology Group (COG) study POG-9481. Blood. 2006;107:4606-4613.

5. Muramatsu H, Kato K, Watanabe N, Matsumoto K, Nakamura T, Horikoshi Y, et al. Risk factors for early death in neonates with Down syndrome and transient leukaemia. Br J Haematol. 2008; 142:610-615.

6. Flasinski M, Scheibke K, Zimmermann M, Creutzig U, Reinhardt K, Verwer F, et al. Low-dose cytarabine to prevent myeloid leukemia in children with Down syndrome: TMD Prevention 2007 study. Blood Adv. 2018;2:1532-1540.

7. Kanda Y. Investigation of the freely available easy-to-use software 'EZR' for medical statistics. Bone Marrow Transplant. 2013;48:452-458

| **Supplementary Table 1.** **Antibodies for the diagnosis of TAM by flow cytometry** | | | |
| --- | --- | --- | --- |
| Specificity | Clone | Fluorochrome | Source |
| CD45 | 2D1 | PerCP | BD Bioscience |
| CD34 | 581 | PE-Cy7 | Beckmann-Coulter |
| CD117 | 104D2D1 | PE-Cy7 | Beckmann-Coulter |
| HLA-DR | Immu-357 | APC | Beckmann-Coulter |
| CD4 | 13B8.2 | FITC | Beckmann-Coulter |
| CD7 | 8H8.1 | PE | Beckmann-Coulter |
| CD56 | N901 | APC | Beckmann-Coulter |
| CD13 | SJ1D1 | PE | Beckmann-Coulter |
| CD33 | D3HL60.251 | APC | Beckmann-Coulter |
| CD36 | FA6.152 | FITC | Beckmann-Coulter |
| CD41 | P2 | FITC | Beckmann-Coulter |
| CD42b | SZ2 | PE | Beckmann-Coulter |
| CD61 | SZ21 | PE-Cy7 | Beckmann-Coulter |

| **Supplementary Table 2. Antibodies for the flow cytometric minimal residual disease measurement** | | | |
| --- | --- | --- | --- |
| Specificity | Clone | Fluorochrome | Source |
| CD36 | CLB-IVC7 | FITC | BD Bioscience |
| CD41 | P2 | FITC | Beckmann-Coulter |
| CD56 | MY31 | PE | BD Bioscience |
| CD42b | SZ2 | PE | Beckmann-Coulter |
| CD34 | 8G12 | PerCP | BD Bioscience |
| CD33 | P67.6 | PE-Cy7 | BD Bioscience |
| CD117 | A3C6E2 | APC | Miltenyi |
| CD45 | 2D1 | APC-H7 | BD Bioscience |
| CD7 | M-T701 | BV421 | BD Bioscience |
| HLA-DR | G46-6 | BV510 | BD Bioscience |

**Supplementary Table 3. Clinical characteristics of TAM patients with or without FCM-MRD samples at 3 months**

|  | Patients with FCM-MRD samples  at 3 months, n = 104 | Patients without FCM-MRD samples at 3 months, n = 46 | *P*-value |
| --- | --- | --- | --- |
| Gender (male:female) | 50:54 | 29:17 | 0.111 |
| Median gestational age, weeks (range) | 37 (31–40) | 37 (29–40) | 0.885 |
| Median birth weight, grams (range) | 2664 (1598–3714) | 2554 (1438–3678) | 0.446 |
| Median age at diagnosis, days (range) | 0 (0–48) | 2 (0–67) | 0.001 |
| Congenital heart disease, n (%) | 73 (70) | 31 (67) | 0.848 |
| Other congenital abnormality | 11(11) | 6(13) | 0.786 |
| Chromosomal status, n |  |  |  |
| Trisomy 21 | 101 | 45 | 1.000 |
| Mosaic trisomy 21 | 1 | 1 | 0.521 |
| Normal karyotype | 2 | 0 | 1.000 |
| Median WBC count at diagnosis, x 10^9^/L (range) | 39.2 (4.7–478.7) | 21.3 (2.4–285.6) | 0.084 |
| Median blasts percentage in PB at diagnosis, % (range) | 34.5 (1–93) | 29.5 (0.5–95.5) | 0.462 |
| Direct bilirubin, mg/dl, median (range) | 0.7 (0.1–12.3) | 0.8 (0–3.0) | 0.356 |
| ALT, IU/L, median (range) | 25 (5–380) | 22 (5–224) | 0.961 |
| Hepatomegaly, cm, median (range)** | 2.5 (0–7) | 2 (0–8) | 0.209 |
| Systemic edema, n (%) | 14 (13) | 3 (7) | 0.273 |
| Organ hemorrhage, n (%) | 6 (6) | 2 (4) | 1.000 |
| Therapeutic interventions |  |  |  |
| Low dose cytarabine, n (%) | 36 (35) | 9 (20) | 0.082 |
| Exchange blood transfusion, n (%) | 9 (9) | 6 (13) | 0.394 |
| Systemic steroid therapy, n (%) | 12 (12) | 7 (15) | 0.597 |
| Classification of *GATA1* mutation |  |  |  |
| High expression type mutation, n (%) | 50 (48) | 14 (30) | 0.050 |
| Low expression type mutation, n (%) | 44 (42) | 22 (48) | 0.594 |
| Unclassified mutation, n (%) | 6 (6) | 10 (22) | 0.008 |
| Negative, n (%) | 3 (3) | 0 (0) | 0.553 |
| Not tested, n (%) | 0 (0) | 0 (0) | - |
| FCM-MRD positivity at 1 month, n (%) | 75/92 (82) | 30/38 (79) | 0.808 |
| Events*, n | 28 | 12 | 1.000 |
| Early death (<9 months of age), n | 2 | 3 | 0.168 |
| Later phase death (after 9 months), n | 6 | 3 | 1.000 |
| Leukemia development, n | 22 | 6 | 0.267 |
| PB, peripheral blood; ALT, alanine transaminase; FCM, flow cytometry; MRD, minimal residual disease | | | |
| *Events were defined by death or leukemia development, **Under costal margin | | | |

| **Supplementary Table 4. Clinical characteristics of 167 TAM patients** | | | | |
| --- | --- | --- | --- | --- |
|  | All patients (N = 167) | Patients with early death (n = 22) | Patients with leukemia development (n = 28) | Patients without events* (n = 110) |
| Gender (male:female) | 89:78 | 13:9 | 14:14 | 59:51 |
| Median gestational age, weeks (range) | 37 (29–40) | 34 (29–39) | 37 (32–40) | 37 (31–40) |
| Median birth weight, grams (range) | 2612 (1066–3714) | 2249 (1066–3044) | 2505 (1838–3444) | 2657 (1598–3714) |
| Median age at diagnosis, days (range) | 1 (0–67) | 0 (0–8) | 1 (0–48) | 1 (0–67) |
| Congenital heart disease, n (%) | 112 (67) | 11 (50) | 21 (75) | 74 (67) |
| Other congenital abnormality | 21 (13) | 4 (18) | 2 (7) | 15 (14) |
| Chromosomal status, n |  |  |  |  |
| Trisomy 21 | 163 | 22 | 27 | 108 |
| Mosaic trisomy 21 | 2 | 0 | 1 | 0 |
| Normal karyotype | 2 | 0 | 0 | 2 |
| Median WBC count at diagnosis, x 10^9^/L (range) | 38.3 (2.4–478.7) | 119.2 (14.3–290.6) | 41.1 (5.8–276.0) | 31.2 (2.4–478.7) |
| Median blasts percentage in PB at diagnosis, % (range) | 37.0 (0.5–95.5) | 60.5 (5–95) | 42 (3–90) | 30 (0.5–95.5) |
| Direct bilirubin, mg/dl, median (range) | 0.7 (0–12.3) | 0.7 (0.2–5.6) | 0.75 (0–8.7) | 0.75 (0–12.3) |
| ALT, IU/L, median (range) | 25 (3–468) | 47 (3–468) | 20 (7–366) | 24.5 (5–38) |
| Hepatomegaly, cm, median (range)** | 3 (0–8) | 5 (0–8) | 2 (0–7) | 2 (0–7) |
| Systemic edema, n (%) | 31 (19) | 15 (68) | 3 (11) | 12 (11) |
| Organ hemorrhage, n (%) | 14 (8) | 6 (27) | 1 (4) | 7 (6) |
| Therapeutic interventions, n (%) | 68 (41) | 15 (68) | 11 (39) | 38 (35) |
| Low dose cytarabine, n (%) | 52 (31) | 9 (41) | 10 (36) | 29 (26) |
| Exchange blood transfusion, n (%) | 20 (12) | 6 (27) | 3 (11) | 11 (10) |
| Systemic steroid therapy, n (%) | 30 (18) | 14 (64) | 0 (0) | 15 (14) |
| Classification of *GATA1* mutation |  |  |  |  |
| High expression type mutation, n (%) | 73 (44) | 10 (45) | 10 (36) | 51 (46) |
| Low expression type mutation, n (%) | 71 (43) | 8 (36) | 14 (50) | 44 (40) |
| Unclassified mutation, n (%) | 19 (11) | 3 (14) | 4 (14) | 12 (11) |
| Negative, n (%) | 3 (2) | 0 | 0 | 3 (3) |
| Not tested, n (%) | 1 (1) | 1 (5) | 0 (0) | 0 (0) |
| FCM-MRD positivity at 1 month, n (%) | 107/133 (80) | 7/8 (88) | 26/27 (96) | 69/92 (75) |
| FCM-MRD positivity at 3 months, n (%) | 20/104 (21) | 1/2 (50) | 9/22 (41) | 10/76 (13) |
| Events*, n | 57 | 22 | 28 | 0 |
| Early death (<9 months of age), n | 22 | 22 | 0 | 0 |
| Later phase death (after 9 months), n | 9 | 0 | 2 | 0 |
| Leukemia development, n | 28 | 0 | 28 | 0 |
| PB, peripheral blood; ALT, alanine transaminase; FCM, flow cytometry; MRD, minimal residual disease | | | | |
| *Events were defined by death or leukemia development, **Under costal margin | | | | |

**Supplementary Table 5. Details and expression type of *GATA1* mutations**

| UPN | *GATA1* mutation | | The number of mutant clones | Mutation Type | Expression type |
| --- | --- | --- | --- | --- | --- |
|  | Nucleotide change | Amino acid change |  |  |  |
| 1 | c.3G>A | - | 1 | LOM | High |
| 2 | c.47_78del32 | p.Pro16Hisfs*13 | 1 | PTC1-5' | Low |
| 3 | c.220+1G>A | - | 1 | SE | High |
| 4 | c.90_91delAG | p.Val32Phefs*7 | 1 | PTC1-5' | Low |
| 5 | not detected | - | 0 | - | - |
| 6 | c.220+1G>A | - | 1 | SE | High |
| 7 | c.115G>T | p.Glu39* | 1 | PTC1-5' | Low |
| 8 | c.90_91delAG | p.Val32Phefs*7 | 1 | PTC1-5' | Low |
| 9 | c.174_175ins14 | p.Ala59Trpfs*83 | 1 | PTC 2 | Low |
| 10 | c.101dupT | p.Ser36Leu*4 | 1 | PTC1-5' | Low |
| 11 | c.1_10del10 | - | 1 | LOM | High |
| 12 | c.212_220del12 | - | 1 | SE | High |
| 13 | c.149_150insA | p.Ser51Glufs*17 | 1 | PTC1-5' | Low |
| 14 | c.49_50delCA | p.Gln17Val*22 | 1 | PTC1-5' | Low |
| 15 | c.[137_138CC>AA;143C>T] | p.Ser46* | 1 | PTC1-5' | Low |
| 16 | c.8dupT | p.Gly5Trpfs*35 | 1 | PTC1-5' | Low |
| 17 | c.173_174ins[T;161_173] | p.Ala59Profs*89 | 1 | PTC 2 | Low |
| 18 | c.162_183delinsAG | p.Ala55Valfs*6 | 1 | PTC1-5' | Low |
| 19 | c.21_221-168del544 | - | 1 | SE | High |
| 20 | c.89C>G | p.Ser30* | 1 | PTC1-5' | Low |
| 21 | c.97_101delTTCTT | p.Phe33Profs*5 | 1 | PTC1-5' | Low |
| 22 | c.83dupC | p.Glu29Argfs*11 | 1 | PTC1-5' | Low |
| 23 | c.205_217del13 | p.Tyr69Glnfs*64 | 1 | PTC 2 | Low |
| 24 | c.149_150delCG | p.Pro50Glnfs*17 | 2 | PTC-1-5' | Multiple mutation |
|  | c.49dupC | p.Gln17Profs*23 |  | PTC-1-5' |  |
| 25 | c.135_153del19 | p.Ser46Glnfs*85 | 1 | PTC 2 | Low |
| 26 | c.140_174del35 | p.Ser47Cysfs*9 | 1 | PTC1-5' | Low |
| 27 | c.165_184dup20 | p.Tyr62Leufs*82 | 2 | PTC2 | Multiple mutation |
|  | c.3G>A | - |  | LOM |  |
| 28 | c.220+2T>C | - | 1 | SE | High |
| 29 | c.231_232dupGT | - | 1 | - | Unclassified |
| 30 | c.153_174dup22 | p.Ala59Hisfs*16 | 1 | PTC1-3' | High |
| 31 | c.149_150insC | p.Ser51Glufs*17 | 1 | PTC1-5' | Low |
| 32 | c.189C>A | pTyr63* | 1 | PTC1-3' | High |
| 33 | c.-19-1G>A | - | 1 | SE | High |
| 34 | c.197_220+1del25 | - | 1 | SE | High |
| 35 | c.220+1G>C | - | 1 | SE | High |
| 36 | c.90_91delAG | p.Val32Phefs*7 | 1 | PTC1-5' | Low |
| 37 | c.89_95dup7 | p.Phe33Argfs*9 | 1 | PTC1-5' | Low |
| 38 | c.166_220+23del78 | - | 1 | SE | High |
| 39 | c.1A>G | - | 2 | LOM | Multiple mutation |
|  | c.121_146del26 | p.Leu41Profs*18 |  | PTC1-5 |  |
| 40 | c.153_154ins10 | pThr52Cysfs*19 | 1 | PTC1-5' | Low |
| 41 | c.-19-1G>A | - | 1 | SE | High |
| 42 | c.220+3delinsCTACAGAC | - | 1 | SE | High |
| 43 | c.-9-285_152del451 | - | 1 | SE | High |
| 44 | c.1A>G | - | 1 | LOM | High |
| 45 | c.220G>T | - | 1 | SE | High |
| 46 | c.18_34del17 | p.Gly7Argfs*27 | 1 | PTC1-5' | Low |
| 47 | c.-19-29_-19-6del24 | - | 1 | SE | High |
| 48 | c.212_220+5del14 | - | 1 | SE | High |
| 49 | c.220+2_220+3insGT | - | 1 | SE | High |
| 50 | c.219A>G | - | 1 | SE | High |
| 51 | c.186C>G | p.Tyr62* | 1 | PTC1-3' | High |
| 52 | not detected | - | 0 | - | - |
| 53 | c.220+1G>A | - | 1 | SE | High |
| 54 | c.182_195dup14 | p.Ala66Profs*76 | 1 | PTC 2 | Low |
| 55 | c.150_160dup11 | p.Thr54Argfs*87 | 1 | PTC 2 | Low |
| 56 | c.150_163deiinsTGGCAGCTGCCC | p.Ser51Glyfs*16 | 1 | PTC1-5' | Low |
| 57 | c.44_48dupTCCCC | p.Gln17Serfs*122 | 1 | PTC 2 | Low |
| 58 | c.174delinsCTACTGGCCGCT | p.Ala59Tyrfs*82 | 1 | PTC 2 | Low |
| 59 | c.220+2T>A | - | 1 | SE | High |
| 60 | c.220+1G>T | - | 1 | SE | High |
| 61 | c.179_182dupTGGC | p.Tyr62Glyfs*7 | 1 | PTC1-3' | High |
| 62 | c.173delC | p.Ala58Glyfs*79 | 1 | PTC 2 | Low |
| 63 | c.1A>T | - | 1 | LOM | High |
| 64 | c.1A>G | - | 1 | LOM | High |
| 65 | c.185_195del11 | p.Tyr62Cysfs*2 | 1 | PTC1-3' | High |
| 66 | c.150delG | p.Ser51Alafs*86 | 1 | PTC 2 | Low |
| 67 | c.-19-160_161del340 | - | 1 | SE | High |
| 68 | c.81_82delAC | p.Pro28Argfs*11 | 1 | PTC1-5' | Low |
| 69 | c.1A>T | - | 1 | LOM | High |
| 70 | c.231_249del19 | - | 2 | - | Multiple mutation |
|  | c.220+3A>C | - |  | SE |  |
| 71 | c.158_173dup16 | p.Ala59Hisfs*14 | 1 | PTC1-3' | High |
| 72 | not detected | - | 0 | - | - |
| 73 | c.161_173dup13 | p.Ala59Argfs*13 | 1 | PTC1-3' | High |
| 74 | c.46_49dupCCCC | p.Glu17Profs*24 | 1 | PTC1-5' | Low |
| 75 | c.[-11_15delinsTCGCAGGTTAA;19G>A] | - | 1 | LOM | High |
| 76 | c.183_186delCTAC | p.Tyr62Thrfs*74 | 1 | PTC 2 | Low |
| 77 | c.140_149dup10 | p.Ser51Hisfs*20. | 1 | PTC1-5' | Low |
| 78 | c.90_91delAG | p.Val32Phefs*7 | 2 | PTC1-5' | Multiple mutation |
|  | c.184_187dupTACT  ) | p.Tyr63Leufs*6 |  | PTC1-3' |  |
| 79 | c.154_162delinsGGTG | p.Thr52Glyfs*14 | 2 | PTC1-5' | Multiple mutation |
|  | c.151_168delinsT | p.Ser51Cysfs*11 |  | PTC-1-5' |  |
| 80 | c.150delG | p.Ser51Alafs*86 | 1 | PTC 2 | Low |
| 81 | c.-19_4del23 | - | 1 | LOM | High |
| 82 | c.219A>G | - | 1 | SE | High |
| 83 | c.1A>G | - | 1 | LOM | High |
| 84 | c.1A>G | - | 2 | LOM | Multiple mutation |
|  | c.174_188dup15 | p.Tyr63* |  | PTC1-3' |  |
| 85 | c.-19-1G>A | - | 1 | SE | High |
| 86 | c.220+1G>C | - | 1 | SE | High |
| 87 | c.5_14del10 | p.Glu2Alafs*132 | 1 | PTC 2 | Low |
| 88 | c.220G>A | - | 1 | SE | High |
| 89 | c.-19-33_-19-15del19 | - | 1 | SE | High |
| 90 | c.-4_8delinsGAC | - | 1 | LOM | High |
| 91 | c.231_232dupGT | - | 1 | - | Unclassified |
| 92 | c.90_91delAG | p.Val32Phefs*7 | 1 | PTC1-5' | Low |
| 93 | c.151_161dup11 | p.Ala56Glnfs*85 | 1 | PTC 2 | Low |
| 94 | c.174_188dup15 | p.Tyr63* | 1 | PTC1-3' | High |
| 95 | c.-19-1G>A | - | 1 | SE | High |
| 96 | c.153-183dup31 | p.Tyr62Hisfs*16 | 1 | PTC1-3' | High |
| 97 | c.189C>A | p.Tyr63* | 1 | PTC1-3' | High |
| 98 | c.90_91delAG | p.Val32Phefs*7 | 1 | PTC1-5' | Low |
| 99 | c.90_91delAG | p.Val32Phefs*7 | 2 | PTC1-5' | Multiple mutation |
|  | c.159_173dup15{insT} | p.Ala59Hisfs*14 |  | PTC1-3' |  |
| 100 | c.174_193dup20 | p.Asp65Glyfs*79 | 1 | PTC 2 | Low |
| 101 | c.154_170dup17 | p.Ala58Glnfs*85 | 1 | PTC 2 | Low |
| 102 | c.121_131del11 | p.Leu41Serfs*23 | 1 | PTC1-5' | Low |
| 103 | c.186C>A | p.Tyr62* | 1 | PTC1-3' | High |
| 104 | c.-19-1G>A | - | 2 | SE | Multiple mutation |
|  | c.181_187dup7{insAAGT} | p.Tyr63* |  | PTC1-3' |  |
| 105 | c.192_193dupGG | p.Asp65Glyfs*73 | 1 | PTC 2 | Low |
| 106 | c.229_357del129 | p.77_119del43 | 1 | - | Unclassified |
| 107 | c.216_220+3delCCCAGGTA | - | 1 | SE | High |
| 108 | c.49C>T | p.Gln17* | 1 | PTC1-5' | Low |
| 109 | c.150_168delinsTGT | p.Ser51Valfs*81 | 1 | PTC 2 | Low |
| 110 | c.124_127dupGATG | p.Ala43Glyfs*26 | 1 | PTC1-5' | Low |
| 111 | c.149_150ins A | p.Ser51Glufs*17 | 2 | PTC1-5' | Multiple mutation |
|  | c.171dupT | p.Ala58Cysfs*10 |  | PTC1-3' |  |
| 112 | c.-19-504_93del616 | - | 1 | SE | High |
| 113 | c.146_173dup28{insCCCCCCC] | p.Ala59Profs*90 | 2 | PTC 2 | Multiple mutation |
|  | c.220+1G>C | - |  | SE |  |
| 114 | not tested | - | - | - | - |
| 115 | c.144_198dup55 | p.Asp65Valfs89* | 1 | PTC 2 | Low |
| 116 | c.105dupC | p.Ser36Leufs*4 | 1 | PTC1-5' | Low |
| 117 | c.220G>A | - | 1 | SE | High |
| 118 | c.220_220+9del10 | - | 1 | SE | High |
| 119 | c.189delC | p.Tyr63* | 1 | PTC1-3' | High |
| 120 | c.-19-3C>G | - | 1 | SE | High |
| 121 | c.108_109delTG | p.Gly37Alafs*2 | 1 | PTC1-5' | Low |
| 122 | c.174_185dup12 | p.Tyr62* | 1 | PTC1-3' | High |
| 123 | c.1A>G | - | 1 | LOM | High |
| 124 | c.220+1G>A | - | 1 | SE | High |
| 125 | c.176_177insTCTGGGC | p.Ala61Glyfs*9 | 1 | PTC1-3' | High |
| 126 | c.90_91delAG | p.Val32Phefs*7 | 1 | PTC1-5' | Low |
| 127 | c.3G>A | - | 1 | LOM | High |
| 128 | c.150_159dup10{insGCAGCTG} | p.Thr54Alafs*89 | 1 | PTC 2 | Low |
| 129 | c.220G>A | - | 1 | SE | High |
| 130 | c.220G>A | - | 1 | SE | High |
| 131 | c.189C>A | p.Tyr63* | 1 | PTC1-3' | High |
| 132 | c.89C>A | p.Ser30* | 1 | PTC1-5' | Low |
| 133 | c.152_153insGTGGGAG | p.Ser51Argfs9* | 1 | PTC1-5' | Low |
| 134 | c.108_177dup70 | p.Leu60Trpfs*3 | 1 | PTC1-3' | High |
| 135 | c.207_208delCA | p.Tyr69* | 1 | PTC1-3' | High |
| 137 | c.183_195del13 | p.Tyr62Leufs*71 | 1 | PTC 2 | Low |
| 138 | c.221-67_433del280 | - | 1 | - | Unclassified |
| 139 | c.90_91delAG | p.Val32Phefs*7 | 1 | PTC1-5' | Low |
| 140 | c.54dupT | p.Val19Cysfs*21 | 1 | PTC1-5' | Low |
| 141 | c.90_91delAG | p.Val32Phefs*7 | 1 | PTC1-5' | Low |
| 142 | c.187dupT | p.Tyr63Leufs*5 | 1 | PTC1-3' | High |
| 143 | c.159_189del31 | p.Thr54Glyfs*73 | 1 | PTC 2 | Low |
| 144 | c.90_91delAG | p.Val32Phefs*7 | 1 | PTC1-5' | Low |
| 145 | c.151_161dup11 | p.Ala56Glnfs*85 | 2 | PTC2 | Multiple mutation |
|  | c.93_94delGG | p.Val32Phefs*7 |  | PTC1-5' |  |
| 146 | c.170_174dupCTGCT | pAla59Leufs*80 | 1 | PTC 2 | Low |
| 147 | c.220+1G>A | - | 1 | SE | High |
| 148 | c.46_49dupCCCC | pGln17Profs*24 | 1 | PTC1-5' | Low |
| 149 | c.150delG | p.Ser51Alafs*86 | 1 | PTC 2 | Low |
| 150 | c.180_187dup8 | p.Tyr63Trpfs*77 | 1 | PTC 2 | Low |
| 151 | c.41_72del32 | p.Pro14Leufs*15 | 1 | PTC1-5' | Low |
| 152 | c.182_189dup8 | p.Arg64Profs*76 | 1 | PTC 2 | Low |
| 153 | c.220+3A>C | - | 1 | SE | High |
| 154 | c.181_185dup5{insGT} | p.Tyr62* | 1 | PTC1-3' | High |
| 155 | c.183_193del11 | p.Tyr62Argfs*2 | 1 | PTC1-3' | High |
| 156 | c.231_241delinsATGGAGGGGA | p.77_119del43 | 1 | - | Unclassified |
| 157 | c.220+1G>A | - | 1 | SE | High |
| 158 | c.38_39delAG | p.Glu13Alafs*26 | 1 | PTC1-5' | Low |
| 159 | c.90_91delAG | p.Val32Phefs*7 | 1 | PTC1-5' | Low |
| 160 | c.348_419delinsCTGACCCTGGGACTC | p.Pro117* | 2 | - | Multiple mutation |
|  | c.187dupT | p.Tyr63Leufs*5 |  | PTC1-3' |  |
| 161 | c.90_91delAG | p.Val32Phefs*7 | 1 | PTC1-5' | Low |
| 162 | c.-19-1G>A | - | 1 | SE | High |
| 163 | c.15_28del14 | p.Leu6Aspfs*29 | 1 | PTC1-5' | Low |
| 164 | c.2T>C | - | 1 | LOM | High |
| 165 | c.157_184dup28 | p.Tyr62Cysfs*15 | 1 | PTC1-3' | High |
| 166 | c.182_195dup14 | p.Ala66Profs*76 | 1 | PTC 2 | Low |
| 167 | c.1A>G | - | 2 | LOM | Multiple mutation |
|  | c.5A>G | p.Glu2Gly |  | - |  |
| 168 | c.81_82delAC | p.Pro28Argfs*11 | 1 | PTC1-5' | Low |
| LOM, loss of the first methionine; PTC, premature termination codon; SE, splicing error | | | | | |

| **Supplementary Table 6. Details of the patients with LDAC** | | | | | |
| --- | --- | --- | --- | --- | --- |
| UPN | WBC,  ×10^9^/L | Duration of LDAC, days | Leukemia development | Grade 4* Adverse events | Survival |
| 1 | 37.5 | 6 | - | B, T, N | Alive |
| 2 | 201.1 | 7 | - | A, H | Alive |
| 4 | 87.8 | 6 | + | N | Alive |
| 6 | 117.3 | 9 | - | - | Alive |
| 8 | 93.7 | 6 | + | E | Alive |
| 9 | 49.7 | 5 | - | N | Alive |
| 17 | 139.6 | 4 | - | - | Alive |
| 19 | 176.0 | 12 | - | N | Alive |
| 26 | 11.1 | 4 | - | N | Alive |
| 33 | 17.6 | 7 | - | A, N | Alive |
| 36 | 93.1 | 8 | - | H, N, T | Alive |
| 40 | 285.6 | 5 | - | N, T | Alive |
| 44 | 114.8 | 7 | - | N | Alive |
| 48 | 25.0 | 1 | - | A, B, H, N | Death |
| 49 | 148.6 | 6 | - | B, C, H, N | Death |
| 50 | 176.0 | 5 | - | B, E, H, N | Death |
| 51 | 39.3 | 7 | - | N | Alive |
| 56 | 76.2 | 3 | - | D | Alive |
| 59 | 45.4 | 3 | - | N | Alive |
| 60 | 90.3 | 6 | - | B, N | Death |
| 62 | 26.5 | 5 | - | A, T | Alive |
| 67 | 119.0 | 7 | - | A, B, F, H, N | Death |
| 69 | 81.2 | 5 | + | - | Alive |
| 74 | 91.9 | 6 | + | N | Alive |
| 76 | 54.4 | 6 | + | T | Alive |
| 77 | 52.1 | 4 | - | D, H | Alive |
| 78 | 175.0 | 6 | - | N | Alive |
| 82 | 107.4 | 6 | - | B, N | Alive |
| 84 | 102.1 | 7 | - | N | Alive |
| 86 | 92.0 | 7 | - | A | Alive |
| 90 | 155.9 | 5 | - | - | Alive |
| 93 | 127.0 | 7 | - | N, T | Alive |
| 94 | 51.4 | 2 | - | H | Alive |
| 96 | 478.6 | 7 | - | A, N, T | Death |
| 98 | 173.4 | 5 | - | N | Alive |
| 100 | 16.1 | 7 | - | N | Death |
| 101 | 53.5 | 3 | - | T | Death |
| 108 | 129.3 | 9 | - | N, T | Alive |
| 112 | 119.4 | 1 | - | N | Death |
| 115 | 15.7 | 7 | - | - | Death |
| 120 | 238.4 | 3 | - | B, H | Death |
| 126 | 27.1 | 5 | - | - | Death |
| 133 | 33.0 | 5 | - | N | Alive |
| 135 | 275.9 | 7 | + | N, T | Alive |
| 137 | 144.7 | 5 | + | - | Alive |
| 145 | 43.1 | 5 | + | - | Alive |
| 147 | 39.1 | 7 | + | A, B | Alive |
| 150 | 35.4 | 3 | + | N | Alive |
| 152 | 16.4 | 7 | - | - | Alive |
| 155 | 174.9 | 7 | - | A, F | Alive |
| 156 | 172.0 | 5 | - | B, E | Death |
| 161 | 87.3 | 4 | - | - | Alive |
| LDAC, low dose cytarabine; B, hyperbilirubinemia; T, thrombocytopenia; N, neutropenia; A, anemia; | | | | | |
| H, hypofibrinogenmia; E, elevated transaminase; C, hypercreatinemia; | | | | | |
| D, disseminated intravascular coagulation; F, febrile neutropenia | | | | | |
| *Adverse events were based on the CTCAE version 3.0. | | | | | |

**Supplementary Table 7. Details of systemic steroid therapy in 30 TAM patients**

| UPN | Type of steroid | Max dosage  , mg/kg | Period of max dosage* , days | Total period of systemic steroid therapy, days | Indication for systemic steroid therapy | Survival |
| --- | --- | --- | --- | --- | --- | --- |
| 1 | PSL | 1 | 21 | 27 | Prevention of liver fibrosis | Alive |
| 3 | HDC | 5 | 12 | 26 | Oliguria | Death |
| 5 | PSL | 1 | 23 | 35 | Prevention of liver fibrosis | Alive |
| 16 | HDC | 24 | 1 | 14 | Circulatory failure | Death |
| 21 | HDC | 10 | 6 | 12 | Prevention of liver fibrosis | Alive |
| 26 | PSL | 2 | 8 | 8 | Chylothorax | Alive |
| 33 | PSL | 1 | 11 | 16 | Prevention of liver fibrosis | Alive |
| 36 | PSL | 2 | 4 | 7 | Hypercytokinemia | Alive |
| 45 | HDC | 4.4 | 1 | 3 | Circulatory failure | Death |
| 48 | PSL | 2 | 3 | 58 | Effusion | Death |
| 49 | mPSL | 4 | 4 | 16 | Circulatory failure | Death |
| 50 | HDC | 100 mg/m2 | 4 | 13 | Hypercytokinemia | Death |
| 58 | PSL | 2 | 6 | 14 | Prevention of liver fibrosis | Alive |
| 63 | HDC | 30 | 1 | 1 | Adrenal insufficiency | Death |
| 67 | PSL | 2 | 9 | 127 | Prevention of liver fibrosis | Death |
| 77 | HDC | 1 | 20 | 56 | Prevention of liver fibrosis | Alive |
| 81 | PSL | 1 | 23 | 44 | Prevention of liver fibrosis | Alive |
| 82 | HDC | 4 | 4 | 18 | Circulatory failure | Alive |
| 84 | DEX | Unknown | 3 | 3 | Unknown | Alive |
| 100 | DEX | 0.2 | 1 | 1 | Prevention of laryngeal edema | Death |
| 101 | HDC | 8 | 1 | 5 | Circulatory failure | Death |
| 108 | PSL | 0.76 | 7 | 14 | Effusion | Alive |
| 114 | HDC | 23 | 7 | 9 | Circulatory failure | Death |
| 115 | HDC | 7 | 4 | 5 | Circulatory failure | Death |
| 123 | HDC | 2.7 | 12 | 22 | Circulatory failure | Alive |
| 132 | HDC | 27 | 1 | 6 | Circulatory failure | Death |
| 138 | mPSL | 10 | 3 | 3 | Circulatory failure | Death |
| 149 | PSL | 2 | 17 | 25 | Anti-leukemic effect | Alive |
| 152 | PSL | 2 | 5 | 8 | Hypercytokinemia | Alive |
| 156 | PSL | 2 | 10 | 10 | Prevention of liver fibrosis | Death |
|  | HDC | 4 | 20 | 20 |  |  |
| *This period does not include tapering period. | | | | | | |
| PSL, prednisolone, HDC, hydorocortisone, DEX, dexamethasone, mPSL, methylprednisolone | | | | | | |

**Supplementary Table 8. Characteristics of TAM patients with and without systemic steroid therapy**

|  | Patients with systemic steroid therapy, n = 30 | Patients without systemic steroid therapy, n = 137 | *P*-value |
| --- | --- | --- | --- |
| Gender (male:female) | 16:14 | 73:64 | 1.000 |
| Median gestational age, weeks (range) | 37 (29–39) | 37 (31–40) | 0.003 |
| Median birth weight, grams (range) | 2492 (1066–3714) | 2637 (1508–3678) | 0.216 |
| Median age at diagnosis, days (range) | 1 (0–6) | 1 (0–67) | 0.058 |
| Congenital heart disease, n (%) | 16 (53) | 96 (70) | 0.089 |
| Other congenital abnormality | 5(17) | 16(12) | 0.542 |
| Chromosomal status, n |  |  |  |
| Trisomy 21 | 30 | 133 | 1.000 |
| Mosaic trisomy 21 | 0 | 2 | 1.000 |
| Normal karyotype | 0 | 2 | 1.000 |
| Median WBC count at diagnosis, x 10^9^/L (range) | 103.4 (4.7–290.6) | 33.0 (2.4–478.7) | <0.001 |
| Median blasts percentage in PB at diagnosis, % (range) | 57.5 (1.3–95) | 31.5 (0.5–95.5) | 0.021 |
| Direct bilirubin, mg/dl, median (range) | 0.9 (0.2–12.3) | 0.7 (0–8.7) | 0.025 |
| ALT, IU/L, median (range) | 66 (5–468) | 23 (3–380) | 0.008 |
| Hepatomegaly, cm, median (range)** | 4 (0–8) | 2 (0–7) | 0.002 |
| Systemic edema, n (%) | 14 (47) | 17 (12) | <0.001 |
| Organ hemorrhage, n (%) | 9 (30) | 5 (4) | <0.001 |
| Therapeutic interventions |  |  |  |
| Low dose cytarabine, n (%) | 17 (57) | 35 (26) | 0.002 |
| Exchange blood transfusion, n (%) | 7 (23) | 13 (9) | 0.057 |
| Classification of *GATA1* mutation |  |  |  |
| High expression type mutation, n (%) | 12 (40) | 61 (45) | 0.689 |
| Low expression type mutation, n (%) | 13 (43) | 58 (42) | 0.411 |
| Unclassified mutation, n (%) | 3 (10) | 16 (12) | 1.000 |
| Negative, n (%) | 1 (3) | 2 (1) | 0.450 |
| Not tested, n (%) | 1 (3) | 0 (0) | 0.180 |
| FCM-MRD positivity at 1 month, n (%) | 16/18 (89) | 91/115 (79) | 0.524 |
| FCM-MRD positivity at 3 months, n (%) | 2/12 (17) | 18/92 (20) | 0.731 |
| Events*, n | 15 | 42 | 0.057 |
| Early death (<9 months of age), n | 14 | 8 | <0.001 |
| Later phase death (after 9 months), n | 1 | 8 | 1.000 |
| Leukemia development, n | 0 | 28 | 0.180 |
| PB, peripheral blood; ALT, alanine transaminase; FCM, flow cytometry; MRD, minimal residual disease | | | |
| *Events were defined by death or leukemia development, **Under costal margin | | | |

| **Supplementary Table 9. Adverse events of 52 patients who received low dose cytarabine (CTCAE ver 3.0)** | | | | | | | | |
| --- | --- | --- | --- | --- | --- | --- | --- | --- |
|  | 0–2 wks (n = 52) | | 3–4 wks (n = 51) | | 5–6 wks (n = 46) | | Total period (n = 52) | |
|  | Grade 3 | Grade 4 | Grade 3 | Grade 4 | Grade 3 | Grade 4 | Grade 3 | Grade 4 |
| Anemia, n (%) | 8 (15) | 4 (8) | 19 (37) | 5 (10) | 8 (17) | 3 (7) | 20 (38) | 9 (17) |
| WBC decreased, n (%) | 1 (2) | 0 | 6 (12) | 1 (2) | 0 | 1 (2) | 6 (12) | 2 (4) |
| Neutrophil count decreased, n (%) | 6 (12) | 7 (13) | 7 (14) | 23 (45) | 3 (7) | 8 (17) | 6 (12) | 28 (54) |
| Platelet count decreased, n (%) | 8 (15) | 16 (31) | 15 (29) | 6 (12) | 7 (15) | 2 (4) | 23 (44) | 11 (21) |
| Fibrinogen decreased, n (%) | 8 (15) | 9 (17) | 4 (8) | 2 (4) | 3 (7) | 1 (2) | 10 (19) | 9 (17) |
| DIC, n (%) | 8 (15) | 2 (4) | 2 (4) | 0 | 2 (4) | 0 | 8 (15) | 2 (4) |
| Creatinine increased, n (%) | 6 (12) | 1 (2) | 3 (6) | 0 | 0 | 0 | 7 (13) | 1 (2) |
| AST increased, n (%) | 6 (12) | 3 (57) | 5 (10) | 2 (4) | 4 (9) | 0 | 9 (17) | 4 (8) |
| ALT increased, n (%) | 7 (13) | 1 (2) | 7 (14) | 1 (2) | 5 (11) | 0 | 12 (23) | 1 (2) |
| Blood bilirubin increased, n (%) | 17 (33) | 7 (13) | 13 (25) | 6 (12) | 10 (22) | 2 (4) | 19 (37) | 10 (19) |
| Nausea, n (%) | 2 (4) | 0 | 3 (6) | 0 | 1 (2) | 0 | 4 (8) | 0 |
| Vomiting, n (%) | 0 | 0 | 1 (2) | 0 | 0 | 0 | 1 (2) | 0 |
| Ileus, n (%) | 0 | 0 | 0 | 0 | 1 (2) | 0 | 1 (2) | 0 |
| Diarrhea, n (%) | 0 | 0 | 1 (2) | 0 | 0 | 0 | 1 (2) | 0 |
| Pancreatitis, n (%) | 0 | 0 | 0 | 0 | 0 | 0 | 0 | 0 |
| Left ventricular systolic dysfunction, n (%) | 8 (15) | 0 | 2 (4) | 0 | 1 (2) | 0 | 9 (17) | 0 |
| Allergic reaction, n (%) | 0 | 0 | 0 | 0 | 0 | 0 | 0 | 0 |
| Febrile neutropenia, n (%)* | 0 | 0 | 1 (2) | 0 | 1 (2) | 0 | 2 (4) | 0 |
| Infection with neutropenia, n (%)** | 2 (4) | 0 | 5 (10) | 0 | 1 (2) | 0 | 7 (13) | 0 |
| Infection, n (%) | 12 (23) | 2 (4) | 13 (25) | 1 (2) | 5 (11) | 0 | 15 (29) | 2 (4) |
| DIC, disseminated intravascular coagulation; AST, aspartate aminotransferase; ALT, alanine transaminase; CTCAE, Common Terminology Criteria for Adverse Events  *Fever of unknown origin, **confirmed infection | | | | | | | | |

**Supplementary Table 10. The cause of death of the 31 patients**

| UPN | Cause of death | Survival time, months | Therapeutic interventions |
| --- | --- | --- | --- |
| 3 | Liver failure | 1 | Steroid |
| 16 | Multiple organ failure | 1 | ET, Steroid |
| 23 | Congenital heart disease | 13 | - |
| 31 | Infection | 9 | - |
| 45 | Liver failure | 0 | Steroid |
| 46 | Respiratory failure | 0 | - |
| 48 | Liver failure | 2 | LDAC, ET, Steroid |
| 49 | Multiple organ failure | 0 | LDAC, Steroid |
| 50 | Multiple organ failure | 0 | LDAC, Steroid |
| 60 | Congenital heart disease | 28 | LDAC |
| 63 | Multiple organ failure | 0 | Steroid |
| 65 | Respiratory failure | 0 | - |
| 67 | Liver failure | 3 | LDAC, Steroid |
| 71 | Leukemia | 46 | - |
| 79 | Multiple organ failure | 0 | ET |
| 80 | Liver failure | 1 | - |
| 92 | Unknown | 6 | ET |
| 96 | Respiratory failure | 22 | LDAC |
| 100 | Congenital heart disease | 12 | LDAC, Steroid |
| 101 | Congenital heart disease | 0 | LDAC, Steroid |
| 112 | Organ hemorrhage | 0 | LDAC |
| 114 | Multiple organ failure | 0 | Steroid |
| 115 | Respiratory failure | 1 | LDAC, Steroid |
| 120 | Disseminated intravascular coagulation | 0 | LDAC, ET |
| 121 | Sudden infant death syndrome | 4 | - |
| 126 | Congenital heart disease | 18 | LDAC |
| 132 | Sepsis | 0 | ET, Steroid |
| 138 | Sepsis | 0 | Steroid |
| 151 | Congenital heart disease | 30 | - |
| 156 | Multiple organ failure | 3 | LDAC, Steroid |
| 167 | Leukemia | 35 | - |
| ET, exchange blood transfusion, LDAC, low dose cytarabine | | | |

| **Supplementary Table 11. Univariable and multivariable analyses of early death** | | | | | | |
| --- | --- | --- | --- | --- | --- | --- |
| Covariates | | Number | Univariable analysis | | Multivariable analysis | |
|  |  |  | HR (95% CI) | *P–value* | HR (95% CI) | *P–value* |
| Gender | Male | 89 | (1) | 0.543 |  |  |
|  | Female | 78 | 0.768 (0.328–1.797) |  |  |  |
| Gestational age | ≥37 weeks | 109 | (1) | <0.001 | (1) | 0.586 |
|  | <37 weeks | 58 | 4.563 (1.860–11.20) |  | 1.390 (0.4252–4.537) |  |
| Birth weight | ≥ 2500 g | 99 | (1) | 0.007 | (1) | 0.037 |
|  | < 2500 g | 68 | 3.439 (1.402–8.438) |  | 3.007 (1.067–8.474) |  |
| Congenital heart disease | No | 55 | (1) | 0.067 |  |  |
|  | Yes | 112 | 0.458 (0.197–1.057) |  |  |  |
| Other congenital abnormality | No | 146 | (1) | 0.400 |  |  |
|  | Yes | 21 | 1.593 (0.539–4.706) |  |  |  |
| Organ hemorrhage | No | 153 | (1) | <0.001 | (1) | 0.184 |
|  | Yes | 14 | 5.711 (2.227–14.64) |  | 2.180 (0.690–6.886) |  |
| Systemic edema | No | 136 | (1) | <0.001 | (1) | <0.001 |
|  | Yes | 31 | 13.21 (5.366–32.53) |  | 13.18 (4.375–39.72) |  |
| WBC | <100x10^9^ /L | 131 | (1) | <0.001 | (1) | 0.002 |
|  | ≥100x10^9^ /L | 36 | 7.956 (3.330–19.01) |  | 6.102 (1.958–19.02) |  |
| Blast % in PB | <37% | 83 | (1) | 0.002 | (1) | 0.212 |
|  | ≥37% | 83 | 6.820 (2.018–23.05) |  | 2.582 (0.582–11.440) |  |
| Type of *GATA1* mutation | High expression | 73 | (1) | 0.782 |  |  |
|  | Low expression | 71 | 0.881 (0.358–2.167) |  |  |  |
| Low dose cytarabine | No | 115 | (1) | 0.318 | (1) | 0.700 |
|  | Yes | 52 | 1.542 (0.659–3.608) |  | 0.793 (0.244–2.579) |  |
| Exchange blood transfusion | No | 147 | (1) | 0.025 | (1) | 0.649 |
|  | Yes | 20 | 2.919 (1.142–7.463) |  | 0.739 (0.201–2.716) |  |
| Systemic steroid therapy | No | 137 | (1) | <0.001 | (1) | 0.007 |
|  | Yes | 30 | 10.28 (4.296–24.58) |  | 4.214 (1.482–11.98) |  |
| Hepatomegaly | <5 cm | 126 | (1) | <0.001 | (1) | 0.787 |
|  | ≥5 cm | 36 | 4.362 (1.851–10.28) |  | 1.178 (0.361–3.847) |  |
| Direct Bilirubin | <5 mg/dL | 150 | (1) | 0.034 | (1) | 0.007 |
|  | ≥5 mg/dL | 4 | 4.915 (1.132–21.33) |  | 10.73 (1.910–60.29) |  |
| AMKL, acute megakaryocytic leukemia; CI, confidence interval; HR, hazard ratio; PB, peripheral blood | | | | | | |

| **Supplementary Table 12. Univariable and multivariable analyses of AMKL development** | | | | |  |  |  |
| --- | --- | --- | --- | --- | --- | --- | --- |
| Covariates | | Number | Univariable analysis | | Multivariable analysis | |  |
|  |  |  | HR (95% CI) | *P–value* | HR (95% CI) | *P–value* |  |
| Gender | Male | 89 | (1) | 0.680 |  |  |  |
|  | Female | 78 | 1.167 (0.558–2.437) |  |  |  |  |
| Gestational age | ≥37 weeks | 109 | (1) | 0.670 |  |  |  |
|  | <37 weeks | 58 | 1.177 (0.559–2.480) |  |  |  |  |
| Birth weight | ≥2500 g | 99 | (1) | 0.530 |  |  |  |
|  | <2500 g | 68 | 1.265 (0.606–2.643) |  |  |  |  |
| Congenital heart disease | No | 55 | (1) | 0.280 |  |  |  |
|  | Yes | 112 | 1.6 (0.686–3.723) |  |  |  |  |
| Other congenital abnormality | No | 146 | (1) | 0.330 |  |  |  |
|  | Yes | 21 | 0.495 (0.122–2.017) |  |  |  |  |
| Organ hemorrhage | No | 153 | (1) | 0.350 |  |  |  |
|  | Yes | 14 | 0.387 (0.053–2.803) |  |  |  |  |
| Systemic edema | No | 136 | (1) | 0.220 |  |  |  |
|  | Yes | 31 | 0.483 (0.149–1.565) |  |  |  |  |
| WBC | <100x10^9^ /L | 131 | (1) | 0.140 |  |  |  |
|  | ≥100x10^9^ /L | 36 | 0.416 (0.128–1.349) |  |  |  |  |
| Blast % in PB | <37% | 83 | (1) | 0.990 |  |  |  |
|  | ≥37% | 83 | 0.994 (0.476–2.074) |  |  |  |  |
| Type of *GATA1* mutation | High expression | 73 | (1) | 0.310 | (1) | 0.440 |  |
|  | Low expression | 71 | 1.519 (0.678–3.406) |  | 1.408 (0.592–3.348) |  |  |
| Low dose cytarabine | No | 115 | (1) | 0.610 |  |  |  |
|  | Yes | 52 | 1.224 (0.569–2.630) |  |  |  |  |
| Exchange blood transfusion | No | 147 | (1) | 0.830 |  |  |  |
|  | Yes | 20 | 0.88 (0.271–2.859) |  |  |  |  |
| Systemic steroid therapy | No | 137 | (1) | <0.001 | (1) | < 0.001 |  |
|  | Yes | 30 | 1/Inf |  | 1/Inf |  |  |
| Hepatomegaly | <5 cm | 126 | (1) | 0.660 |  |  |  |
|  | ≥5 cm | 36 | 0.802 (0.304–2.118) |  |  |  |  |
| Direct bilirubin | <5 mg/dL | 150 | (1) | 0.750 |  |  |  |
|  | ≥5 mg/dL | 4 | 1.35 (0.209–8.731) |  |  |  |  |
| FCM-MRD at 1 month | Negative | 26 | (1) | 0.056 |  |  |  |
|  | Positive | 107 | 7.059 (0.951–52.4) |  |  |  |  |
| FCM-MRD at 3 months | Negative | 84 | (1) | 0.002 | (1) | < 0.001 |  |
|  | Positive | 20 | 3.901 (1.631–9.332) |  | 4.709 (1.936–11.450) |  |  |
| AMKL, acute megakaryocytic leukemia; CI, confidence interval; HR, hazard ratio; PB, peripheral blood; FCM, flow cytometry; MRD, minimal residual disease | | | | | | |  |
|  | | | | | | |  |

| **Supplementary Table 13. Rates of early death and leukemia development in patients with TAM** | | | | | | | | |
| --- | --- | --- | --- | --- | --- | --- | --- | --- |
|  | Number of patients | Median WBC count, x 10^9^/L | LDAC, n (%) | ET, n (%) | Steroid,  n (%) | Early death | | Leukemia development, n (%)* |
|  |  |  |  |  |  | n | % (95% CI) |  |
| Massey, et al. (2006) | 48 | 28.8 | 0 (0) | 2 (4) | ND | 8 | 17 (7.5–30.2) | 9 (22) |
| Muramatsu, et al. (2008) | 70 | 49.3 | 0 (0) | 14 (20) | 9 (13) | 16 | 23 (13.7–34.4) | 12 (22) |
| Gamis, et al. (2011) | 135 | 32.8 | 24(18) | 10 (7) | ND | 24 | 18 (11.7–25.3) | 21 (20) |
| Klusmann, et al. (2008) | 146 | 40.3 | 28 (19) | ND | ND | 22 | 15 (9.7–21.9) | 29 (23) |
| TAM-10 study | 167 | 38.3 | 52 (31) | 20 (12) | 31 (19) | 22 | 13 (8.4–19.3) | 28 (20) |
| Flasinski, et al. (2018) | 102 | 24.0 | 39 (38) | ND | ND | 5 | 5 (1.6–11.1) | 17 (17) |
| LDAC, low dose cytarabine; ET, exchange blood transfusion; CI, confidence interval; ND, not described | | | | | | | | |
| *Leukemia development percentage are calculated except patients with death event before leukemia development. | | | | | | | | |


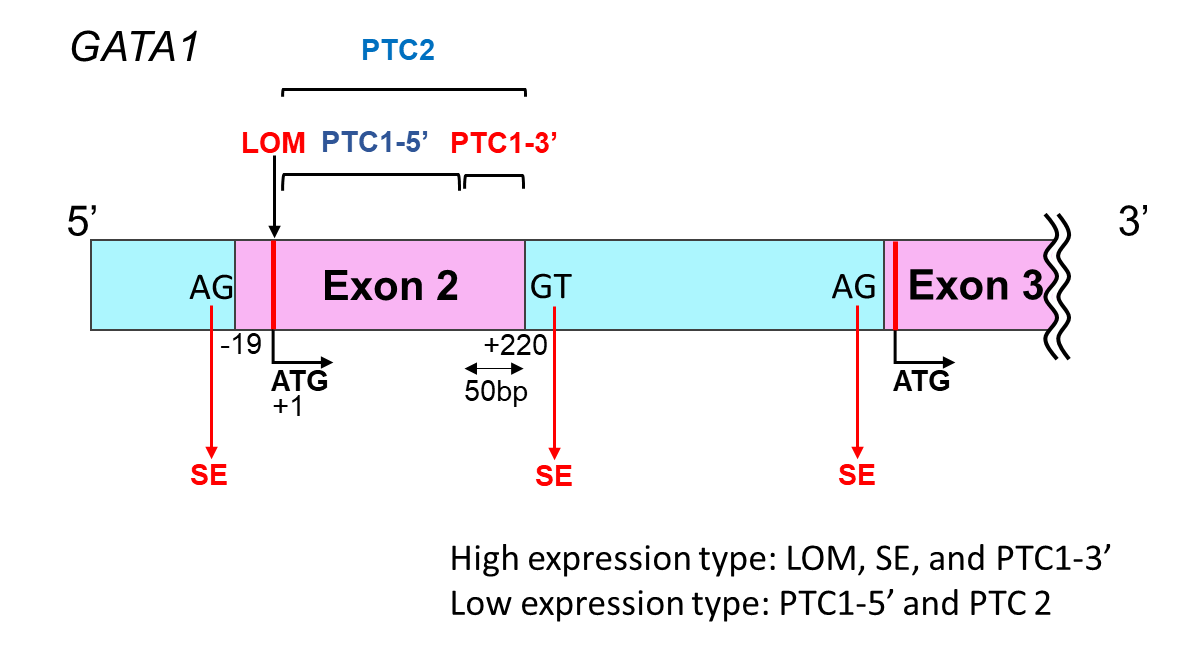


**Supplementary Figure 1. Details of expression type in *GATA1* mutations**

Mutant *GATA1* genes affected the expression levels of the translation products. The transcripts coding the GATA1s protein were categorized into three groups as follows: loss of the first methionine (LOM), splicing error (SE), and premature termination codon (PTC) based on findings in a previous study (Kanezaki, 2010). In addition, the PTC group was classified into two subcategories according to the location of the introduced PTC. A mutation that causes a PTC before the second methionine at codon 84 was classified as PTC type 1 and a mutation after codon 84 was classified as PTC type 2. Thus, PTC1 and PTC2 introduce the premature termination codon (PTC) before and after the second translation start codon (Met84), respectively. Furthermore, PTC type 1 consists of PTC type 1-3’ and PTC type 1-5’. PTC type 1-3’ indicates that the GATA1 mutations are located on the 3’ side of exon 2 (+169 to +218 in the mRNA from the ATG translation initiation codon). PTC type 1-5’ indicates that the mutations are located on the 5’ side of exon 2. The high expression type mutation was defined by LOM, SE, and PTC1-3’. The low expression type mutation was defined by PTC1-5’ and PTC 2.


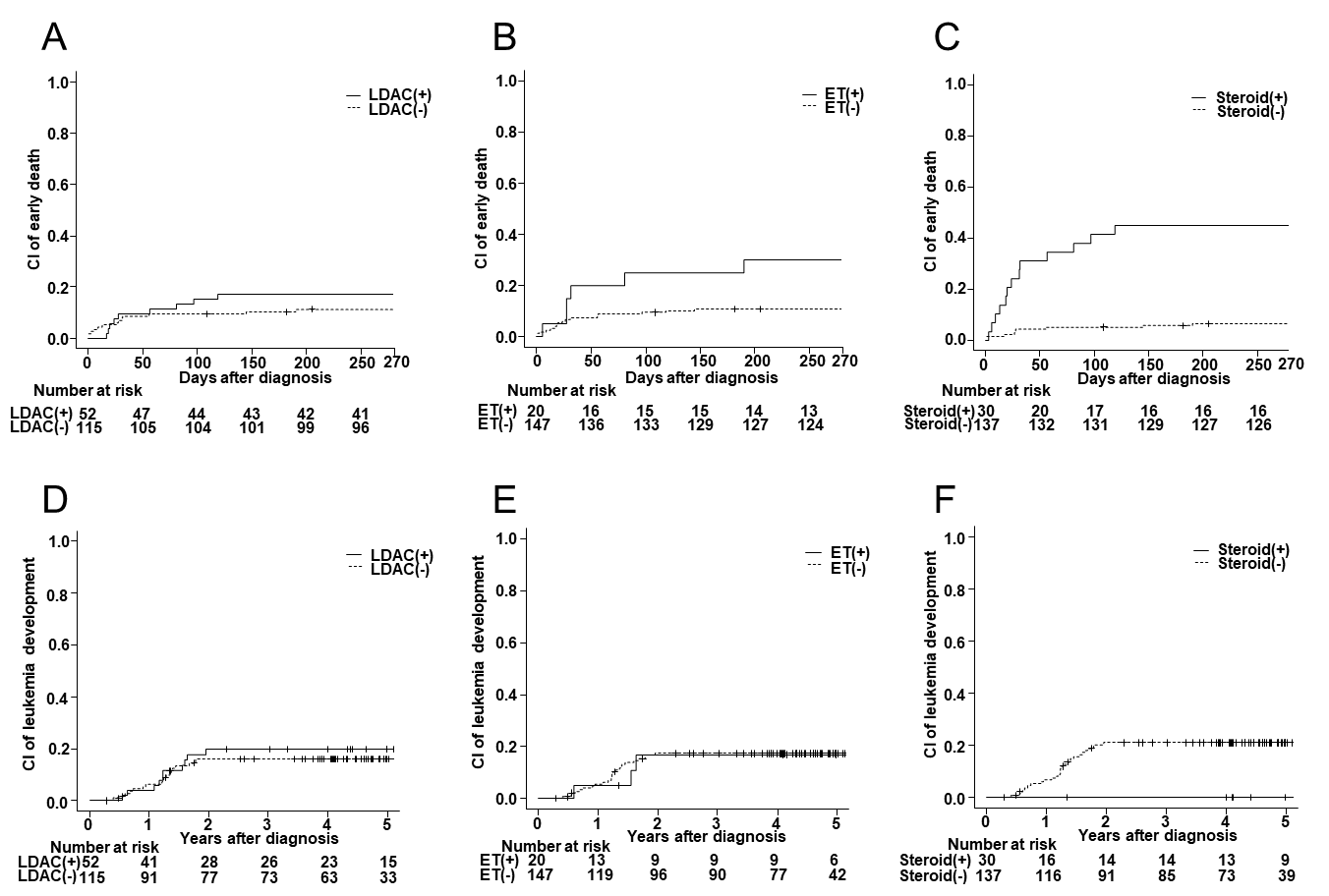


**Supplementary Figure 2. Cumulative incidence rates of early death and leukemia development between patients with or without therapeutic intervention**

This figure shows the cumulative incidence (CI) rates of early death and leukemia development between patients with or without therapeutic intervention. (A), (B) There was no significant difference in the CI of early death between patients with or without LDAC or ET. (C) However, systemic steroid therapy was associated with a higher CI rate of early death (*P* < 0.001). (D), (E) LDAC and ET did not reduce the CI of leukemia development; (F) however, systemic steroid therapy significantly reduced

leukemia development [CIR (95% CI), 0% vs. 21.1% (14.6%–28.4%), *P* = 0.008).


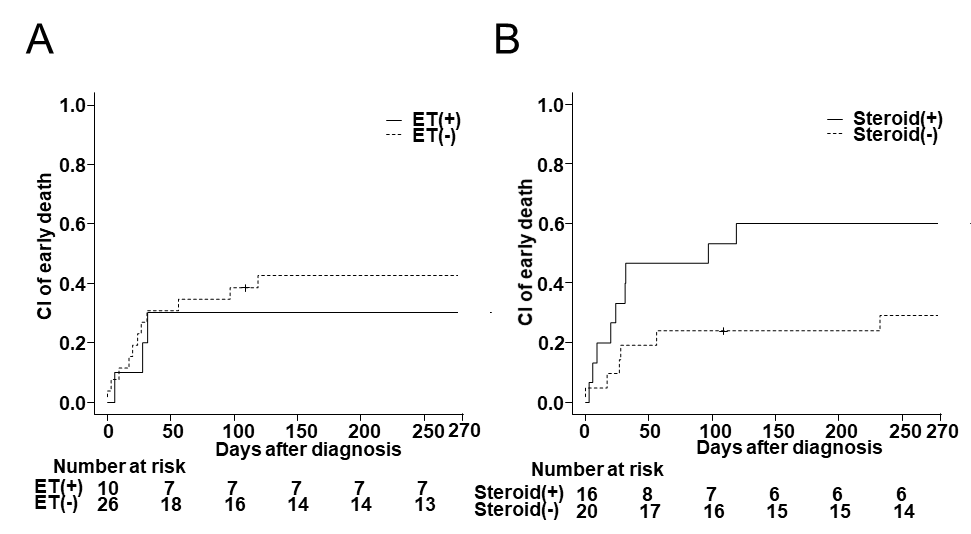


**Supplementary Figure 3. Subgroup analysis in 36 patients with a high WBC count (≥100 × 10^9^ cells/L)**

(A), (B) There was no significant difference in the cumulative incidence rate (CI) of early death between patients with or without ET or steroid therapeutic interventions. ET, exchange blood transfusion
